# Usher syndrome type IV: clinically and molecularly confirmed by novel *ARSG* variants

**DOI:** 10.1007/s00439-022-02441-0

**Published:** 2022-02-28

**Authors:** Hedwig M. Velde, Janine Reurink, Sebastian Held, Catherina H. Z. Li, Suzanne Yzer, Jaap Oostrik, Jack Weeda, Lonneke Haer-Wigman, Helger G. Yntema, Susanne Roosing, Laurenz Pauleikhoff, Clemens Lange, Laura Whelan, Adrian Dockery, Julia Zhu, David J. Keegan, G. Jane Farrar, Hannie Kremer, Cornelis P. Lanting, Markus Damme, Ronald J. E. Pennings

**Affiliations:** 1 Hearing and Genes, Department of Otorhinolaryngology, Radboudumc, Nijmegen, The Netherlands; 2 Donders Institute for Brain, Cognition and Behaviour, Radboudumc, Nijmegen, The Netherlands; 3 Department of Human Genetics, Radboudumc, Nijmegen, The Netherlands; 4 Department of Biochemistry, University of Kiel, Kiel, Germany; 5 Department of Ophthalmology, Radboudumc, Nijmegen, The Netherlands; 6 Eye Center, Medical Center - University of Freiburg, Faculty of Medicine, University of Freiburg, Freiburg, Germany; 7 The School of Genetics and Microbiology, Smurfit Institute of Genetics, Trinity College Dublin, Dublin, Ireland; 8 Next Generation Sequencing Laboratory, Pathology Department, The Mater Misericordiae University Hospital, Dublin, Ireland; 9 Mater Clinical Ophthalmic Genetics Unit, The Mater Misericordiae University Hospital, Dublin, Ireland

## Abstract

Usher syndrome (USH) is an autosomal recessively inherited disease characterized by sensorineural hearing loss (SNHL) and retinitis pigmentosa (RP) with or without vestibular dysfunction. It is highly heterogeneous both clinically and genetically. Recently, variants in the arylsulfatase G (*ARSG*) gene have been reported to underlie USH type IV. This distinct type of USH is characterized by late-onset RP with predominantly pericentral and macular changes, and late onset SNHL without vestibular dysfunction. In this study, we describe the USH type IV phenotype in three unrelated subjects. We identified three novel pathogenic variants, two novel likely pathogenic variants, and one previously described pathogenic variant in *ARSG*. Functional experiments indicated a loss of sulfatase activity of the mutant proteins. Our findings confirm that *ARSG* variants cause the newly defined USH type IV and support the proposed extension of the phenotypic USH classification.

**Supplementary Information:**

The online version contains supplementary material available at 10.1007/s00439-022-02441-0.

## Introduction

Usher syndrome (USH) is an autosomal recessively inherited disease characterized by sensorineural hearing loss (SNHL) and retinitis pigmentosa (RP) with or without vestibular dysfunction. It is the leading cause of inherited deaf-blindness with an estimated prevalence of 3–17 per 100,000 individuals (Boughman et al. 1983; Grondahl 1987; Hallgren 1959; Hope et al. 1997; Kimberling et al. 2010; Rosenberg et al. 1997; Spandau and Rohrschneider 2002). USH is highly heterogeneous from both a clinical and a genetic perspective. Understanding the different genotypes and associated phenotypes is essential for genetic counseling and informing patients about their prognosis. It is also necessary to evaluate the efficacy of future therapies and to select patients that are eligible for such therapies.

Previously, three clinical types of USH were distinguished based on the age of onset, severity, and progression of symptoms (Davenport and Omenn 1977; Smith et al. 1994). USH type I is characterized by severe to profound congenital SNHL, the onset of RP in the first decade of life, and vestibular areflexia (Nisenbaum et al. 2021). USH type II is the most common type of the disease, with moderate to severe congenital SNHL and onset of RP in the second decade of life or later (Nisenbaum et al. 2021; Tsilou et al. 2002). Vestibular function was previously considered unaffected. However, recent studies have also identified vestibular defects in USH II patients (Magliulo et al. 2017; Stemerdink et al. 2021; Wafa et al. 2021). USH type III displays variable progressive SNHL with the age of onset usually in the first decade of life, variable onset of RP, typically by the second decade of life, and variable vestibular function (Geng et al. 2017; Nisenbaum et al. 2021). Nine USH genes have so far been identified: *MYO7A* (OMIM 276900 (Weil et al. 1995)), *USH1C* (OMIM 276904 (Verpy et al. 2000)), *CDH23* (OMIM 601067 (Bolz et al. 2001; Bork et al. 2001)), *PCDH15* (OMIM 602083 (Ahmed et al. 2001; Alagramam et al. 2001)), *USH1G* (OMIM 606943 (Weil et al. 2003)) (type I); *USH2A* (OMIM 276901 (Eudy et al. 1998; Van Wijk et al. 2004)), *ADGRV1* (OMIM 605472 (Weston et al. 2004))*, WHRN* (OMIM 611383 (Ebermann et al. 2007)) (type II); and *CLRN1* (OMIM 276902 (Sankila et al. 1995)) (type III). *CIB2* (OMIM 605564) was described to be associated with USH type I but this was later refuted (Booth et al. 2018; Riazuddin et al. 2012). Finally, *PDZD7* (OMIM 612971) has been suggested to be a modifier gene of retinal disease and a contributor to digenic USH (Ebermann et al. 2010) and was shown to be causal to non-syndromic hearing loss type DFNB57 (OMIM 618003 (Booth et al. 2015; Vona et al. 2016)).

In 2018, a homozygous missense variant (Supplemental Table 1) in the arylsulfatase G (*ARSG*) gene (OMIM 610008) was associated with an atypical USH phenotype characterized by late-onset RP with a distinctive retinal phenotype of ring-shaped atrophy along the arcades, late-onset progressive SNHL, and no vestibular involvement (Khateb et al. 2018). Later, when a second homozygous *ARSG* missense variant was described that caused a similar remarkably late onset of visual and hearing impairment, this phenotype was defined as USH type IV (OMIM 618144) (Abad-Morales et al. 2020). The classification as a fourth, distinct USH type was confirmed by the identification of three additional variants associated with similar phenotypic characteristics (Peter et al. 2020). Five other novel *ARSG* variants were subsequently reported to underlie this newly described phenotype (Fowler et al. 2021; Igelman et al. 2021).

*ARSG* encodes the sulfatase ARSG, which is transported to lysosomes after biosynthesis in the endoplasmic reticulum (ER) (Ferrante et al. 2002; Kowalewski et al. 2014). ARSG has been shown to hydrolyze pseudosubstrates such as *p*-nitrocatechol sulfate (pNCS) and 4-methylumbelliferyl sulfate (Frese et al. 2008). Moreover, the physiological substrate of ARSG has been determined to be 3-*O*-sulfated glucosamine in heparan sulfate and ARSG was shown to be critical for the complete lysosomal degradation of 3-*O*-glucosamine modified heparan sulfate (Kowalewski et al. 2014). The ARSG sulfatase is ubiquitously expressed in mice, including the kidney, testis, liver, spleen, brain, retina, and cochlea (Girotto et al. 2014; Kowalewski et al. 2014; Kowalewski 2015; Kruszewski et al. 2016; Ratzka 2010).

This report confirms both clinically and molecularly that *ARSG* variants are causal to USH type IV. We describe three novel pathogenic *ARSG* variants, two novel likely pathogenic variants, and one previously described pathogenic variant in three unrelated individuals. All subjects share a phenotype of late-onset SNHL and RP, characterized by predominantly pericentral and macular changes.

## Materials and methods

For this retrospective case study, clinical and genetic data of included subjects were obtained from the Radboud university medical center (Nijmegen, the Netherlands), the University Medical Center Freiburg (Germany) and the Mater Misercordiae University Hospital (Dublin, Ireland). All included subjects were previously seen at the out-patient clinic of these hospitals.

### Clinical evaluation of recruited subjects

To obtain a complete description of the subjects' phenotypes, we retrospectively collected demographic data and general medical history, ophthalmic data, audiological data, and data on vestibular function. Demographics and medical history included age, descent, comorbidities, medication use, and medical family history. For ophthalmic phenotyping, we collected ocular history, age of onset and progression of ophthalmic symptoms, best corrected visual acuity, refractive error, slit lamp examination findings, and multimodal imaging including color fundus photography, optical coherence tomography (OCT), visual field tests, electroretinography (ERG), and fundus autofluorescence. Audiological data included age of onset, type and progression of audiological symptoms, medical history of the ear, otoscopic assessment, pure tone and speech audiometry, and speech-in-noise testing. The medical history of the ear included recurrent ear infections, previous noise exposure, the use of ototoxic medication, meningitis, severe head injuries, and ear surgeries. Finally, we collected data on vestibular function by identifying whether vestibular symptoms were present and whether vestibular function had been examined. Incomplete data were supplemented with additional diagnostics if possible.

### Auditory phenotype analyses

RStudio (version 1.4.1106) was used to analyze and plot the thresholds for pure-tone audiometry. Air conduction thresholds were used to analyze the observed severity and degree of symmetry of hearing loss and audiogram configuration. In case of an air–bone gap, the bone conduction thresholds of all audiograms of the subject in question were used instead. In addition, age-related typical audiograms (ARTA) were derived from linear regression analysis of all available audiometric data of included and previously reported subjects as described to provide insight into the rate of progression of hearing loss (Huygen et al. 2003). In addition to assessing potential differences in the progression of hearing loss, the age of onset of hearing loss was determined. The annual threshold deterioration was calculated using cross-sectional non-linear regression analyses of hearing thresholds as a function of age. First, the pure-tone average (PTA) across 0.5–4.0 kHz (PTA_0.5–4 kHz_) for each subject was determined. A two-parameter logistic function was used to fit the data, i.e. ${\mathrm{PTA}}_{0.5-4 \mathrm{kHz}} \sim \frac{130}{1+{e}^{\left(-scale* \left(age-{age}_{mid}\right)\right)}}$, similar to what has been described previously (Pauw et al. 2011). The parameter *age*_*mid*_ describes the age at which the hearing thresholds are halfway between 0 dB and the asymptotic value (fixed at 130 dB). The parameter *scale* represents the slope of the function at this midpoint, which was used as an estimate for the annual threshold deterioration. The calculated age of onset was defined as when the calculated hearing threshold exceeds 25 dB, defined by the WHO as abnormal hearing, and subjects could benefit from amplification (Informal Working Group on Prevention of Deafness and Hearing Impairment Programme Planning & World Health Organization 1991). The calculated age of onset was determined by interpolating the function fit using the parameters *age*_*mid*_ and *scale.* For comparison, the age of onset of SNHL for USH IIa was calculated the same way based on previously published data (Hartel et al. 2016).

### Genetic analyses

For subject N, exome sequencing with targeted gene panels for vision disorders (version DG-2.13, consisting of 408 genes) and hearing impairment (version DG-2.15 and DG-2.18, consisting of 173 and 208 genes) was performed in the ISO15189 accredited Genome Diagnostic Laboratory of the Radboud university medical center (Nijmegen, the Netherlands) according to routine diagnostic procedures (Haer-Wigman et al. 2017). Variants in genes from these panels with a frequency below 5% in the dbSNP database and below 1% in an in-house database (consisting of exome sequencing data of 24,488 individuals mainly of Dutch origin) and within the exon or within the intronic position of −8 to + 3 were interpreted and classified based on existing variant classification guidelines established by the Association for Clinical Genetic Science (Plon et al. 2008; Wallis et al. 2013).

Subject D and F were pre-screened to exclude biallelic variants in other USH-associated genes in Trinity College Dublin (Ireland) as part of the Target 5000 study (Whelan et al. 2020) (subject D) and in the University Medical Center Freiburg (Germany) with the 2016 CeGat panel for USH (CeGaT GmbH, Tübingen, Germany) (subject F). The CeGat panel for subject F was later expanded to include *ABCA4* (OMIM 601691), *CNGA1* (OMIM 123825), *EYS* (OMIM 612424), *PDE6A* (OMIM 180071), *PDE6B* (OMIM 180072), and *RPE65* (OMIM 180069)*.*

Genome sequencing was performed for subjects D and F as described before, with 2 × 150nt paired-end reads and a minimal median coverage of 30 × (Fadaie et al. 2021). In the analysis of genome sequencing data, single nucleotide variants were first prioritized based on the in-house frequency (< 1%, database consisting of genome sequencing data of 702 individuals mainly of Dutch origin) and the Genome Aggregation Database (gnomAD) minor allele frequency (≤ 1%) (Karczewski et al. 2020). All exonic variants, variants at exon–intron boundaries, and deep-intronic variants in genes associated with USH were considered and ranked based on predicted pathogenicity. All stop-gain variants were considered pathogenic. Missense variants were evaluated using the following prediction tools and threshold scores: CADD_PHRED (≥ 15) (Kircher et al. 2014), PhyloP (≥ 2.7) (Pollard et al. 2010), Grantham (≥ 80) (Grantham 1974), SIFT (Ng and Henikoff 2001), and MutationTaster (Schwarz et al. 2014). Potential effects on splicing were evaluated by employing SpliceAI (≥ 0.1/1) (Jaganathan et al. 2019). Copy number variants (detected with Control-FREEC Copy number and allelic content caller (Boeva et al. 2012)) and structural variants (detected with Manta Structural Variant Caller (Chen et al. 2016)) were prioritized based on whether they passed our in-house quality filter and whether they affected genes known to be associated with USH. All variants were visualized with Integrative Genomics Viewer (version 2.4) to confirm their presence.

When DNA of relatives was available or variants were in close proximity to each other, the presence of variants *in trans* was determined by PCR and subsequent Sanger sequencing according to standard protocols.

### Functional analyses of identified variants

Immunoblot and sulfatase activity assays were performed as previously described (Khateb et al. 2018; Peter et al. 2020). Therefore, cDNA expression vectors coding for C-terminally 3xFLAG-tagged wildtype (WT) and mutated ARSG were generated by PCR and cloning into the pcDNA4/TO-C-3xFlag vector with *Eco*R1 and *Xho*I followed by transfection and selection of stable cell lines (primer sequences are provided in Supplemental Table 2). Both patient-derived missense variants were introduced by site-directed mutagenesis as previously described in the WT *ARSG* (Zheng et al. 2004) (primer sequences are provided in Supplemental Table 2). All constructs were sequence-validated by Sanger sequencing. Subsequently, HT1080 cells were transfected as previously described (Peter et al. 2020). HT1080 cells were treated with 600 µg/ml Zeocin (Invivogen, San Diego, CA, USA) three days after transfection, and stable cell lines were used for immunoblotting and activity assays after reaching confluence and passaging in Zeocin-containing selection medium after two passages. Immunoblotting of transfected HT1080 cells was performed according to standard procedures with a nitrocellulose membrane and antibodies against the FLAG-tag (mouse monoclonal, clone M2; Sigma Aldrich, St. Louis, MO, USA), as well as glyceraldehyde 3-phosphate dehydrogenase (rabbit polyclonal, SC-335; Santa Cruz Biotechnology, Dallas, TX, USA). Sulfatase activity was measured photometrically by the turnover of pNCS from protein lysates of stable HT1080 cell lines with the WT *ARSG* construct or the construct harboring an *ARSG* variant.

Minigene splice assays were performed for variants with a predicted effect on splicing. The genomic regions of interest, including the exon and 500nt intron sequence at both ends, were amplified from patient DNA with PCR (primer sequences are provided in Supplemental Table 2). The PCR product was then inserted in a pCI-NEO vector, adapted to the Gateway® cloning system as previously described (Sangermano et al. 2016). The vector inserts were verified by Sanger sequencing. WT and mutant constructs (500 ng) were subsequently transfected individually into HEK293T cells using polyethylenimine (100 µg/ml in 150 mM NaCl). Approximately 24 h post-transfection, RNA was isolated by employing the NucleoSpin RNA Clean-up Kit (Macherey-Nagel, Düren, Germany). 500 ng of RNA was used for cDNA synthesis with the iScript cDNA synthesis kit (Bio-Rad, Hercules, CA, USA), following the manufacturer's instructions. The cDNA derived from the vector insert was amplified with primers for RHO exons 3 and 5 and amplicons were screened with Sanger sequencing. A PCR for *GAPDH* was performed as cell lysis control.

## Results

### Clinical evaluation of recruited subjects

Subject N is a 51-year-old Dutch man of Turkish descent seen at the Radboud university medical center in Nijmegen, the Netherlands. His medical history was uneventful, besides hypothyroidism and occupational noise exposure for many years. In particular, there was no relevant ophthalmic history nor any family history related to hearing or vision loss. His parents were not known to be consanguineous. He experienced peripheral vision loss and night blindness since the age of 46 and was diagnosed with RP aged 49 (Supplemental Table 3). His visual acuity was relatively spared (OD (right eye) 20/25 and OS (left eye) 20/32 Snellen). Ophthalmoscopy revealed waxy pallor of the optic disc, mild attenuation of the vessels, and a preserved fovea with perifoveal retinal pigment epithelial mottling. Atrophy was present around the vascular arcades; adjacent to the atrophy, predominantly located nasally and superior to the optic disc, intraretinal bone spicules were noted (Supplemental Fig. 1). The far periphery appeared relatively preserved. OCTs showed foveal sparing with only paracentral loss of the outer retina, corresponding to the relatively mild loss of visual acuity. On fundus autofluorescence, a ring of hyperautofluorescence was visible in the posterior pole at the transition zone. The mid-periphery showed confluent areas of hypoautofluorescence with preserved autofluorescence in the far periphery. ERG revealed lower scotopic than photopic responses; both were diminished. Goldmann perimetry showed an absolute ring scotoma with intact central vision and a relatively preserved peripheral field in accordance with a normal V-4e Isopter. At the age of 42, before his RP diagnosis, subject N was diagnosed with bilateral SNHL for which he is rehabilitated with bilateral conventional hearing aids (Supplemental Table 4). In addition, he experienced tinnitus. Physical examination and otoscopy showed no abnormalities. Pure tone audiometry revealed bilateral moderate to severe SNHL, with air and bone conduction PTAs (0.5–4.0 kHz) of 49 and 46 dB hearing level (dB HL) (right) and 55 and 53 dB HL (left) at age 46. At age 49, there was asymmetry in the air conduction thresholds, mainly in the high frequencies to the detriment of the right. An air–bone gap was observed primarily in the right ear, which could not be explained by otoscopy or CT. The latter revealed no congenital temporal bone abnormalities. At that time, air and bone conduction PTAs were 70 and 51 dB HL (right) and 64 and 55 dB HL (left). Based on the bone conduction PTAs, the progression in three years was 5 (right) and 3 dB HL (left). The subject did not report any vestibular problems.

Subject F is a 72-year-old woman of Caucasian origin, recruited at the Eye Center of the University of Freiburg, Germany. She was diagnosed with hypothyroidism earlier in life. There was no relevant medical or family history related to hearing, vision, or vestibular function. Her parents were not known to be consanguineous. This individual experienced central vision loss from the age of 40 (Supplemental Table 3). Visual acuity deteriorated to hand movement vision only. Ophthalmoscopy revealed a pale optic disc, mild attenuation of the vessels, and atrophy around the arcades with intraretinal bone spicules predominantly located nasally and superior to the atrophy. The far periphery appeared intact. OCT showed complete loss of the outer retinal layers. Fundus autofluorescence showed extensive hypoautofluorescence in the periphery that extended to the inner part of the vascular arcades, leaving a small ring of relative normal autofluorescence in the posterior pole. ERG revealed absence of both cone and rod responses. As the disease progressed, concentric restriction of the peripheral visual field and a central scotoma developed. Subject F also experienced progressive, bilateral SNHL since the age of 20 for which she has been using bilateral conventional hearing aids since age 31–40 (Supplemental Table 4). At the age of 60 and 72, pure-tone audiometry showed moderate to severe SNHL with a flat audiogram configuration and PTAs of 73 dB HL (both ears) and 90 (right) and 85 dB HL (left). There were no additional problems, notably no reported vestibular symptoms. Bilateral bithermal vestibular caloric stimulation revealed a symmetrical response of the lateral semicircular canals.

Subject D is an 87-year-old man of Caucasian origin seen at the Mater Misercordiae University Hospital in Dublin, Ireland. He had pulmonary tuberculosis when he was 19 years old. Later in life, he developed gastro-esophageal reflux disease, atrial fibrillation, and benign prostatic hyperplasia. This subject was diagnosed with exotropia of the left eye in his early youth. When he was over 60 years old, a posterior subcapsular cataract was observed bilaterally. Medical and family history was uneventful, his parents were not known to be consanguineous. At the age of 51–60, he experienced night blindness and photophobia (Supplemental Table 3). Over time, visual acuity deteriorated to light perception in the right eye and no-light perception in the left eye at age 86. Retinal imaging revealed excavation of the nerve heads, mild attenuation of the vessels, and central atrophy connecting to atrophy around the vascular arcades. This was surrounded by a ring of intraretinal bone-spicule hyperpigmentation, nasally and superior to the disc. Fundus autofluorescence showed central hypoautofluorescence and large confluent areas of hypoautofluorescence adjacent and posterior to the vascular arcades. OCT revealed complete loss of outer retinal layers. At the age of 31–40, subject D developed bilateral SNHL for which he was rehabilitated by bilateral conventional hearing aids (Supplemental Table 4). No other audiological data were available or could be obtained. He reported no vestibular symptoms.

**Fig. 1:**
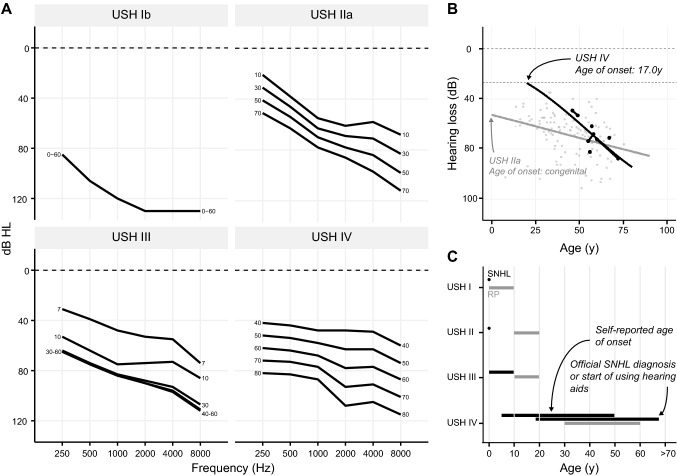
Audiologic features of USH IV compared to other USH types.** A** ARTA of USH IV compared to USH Ib (Wagenaar et al. 1999), USH IIa (Hartel et al. 2016), and USH III (Plantinga et al. 2004). Age (in years) is indicated at both ends of all lines. **B** Calculated age of onset of SNHL for USH IV based on individual PTA_0.5–4 kHz_ and compared to USH IIa (Hartel et al. 2016). Black dots represent USH IV PTA_0.5–4 kHz_ data; gray dots represent USH IIa PTA_0.5–4 kHz_ data, connected dots represent longitudinal data when more than one audiogram was available for one subject. **C** Visualization of age of onset of RP (in gray) and SNHL (in black) of USH IV compared to published data for other USH types (Geng et al. 2017; Millan et al. 2011; Nisenbaum et al. 2021; Tsilou et al. 2002) (Table 2). *dB HL* decibel hearing level; *RP* retinitis pigmentosa; *SNHL* sensorineural hearing loss; *y* years

Audiograms of the right and left ears of included and previously reported subjects showed a predominantly bilateral moderate to severe high-frequency SNHL (Supplemental Fig. 2). The ARTA displayed an average progression of hearing loss of 1.0–1.5 dB HL per year (Fig. 1). This corresponds with the mean progression of 1.25 dB HL per year of both included subjects with more than one audiogram available (subjects N and F). The calculated age of SNHL onset was 17 years (Fig. 1).

### Identification of *ARSG* variants

**Fig. 2:**
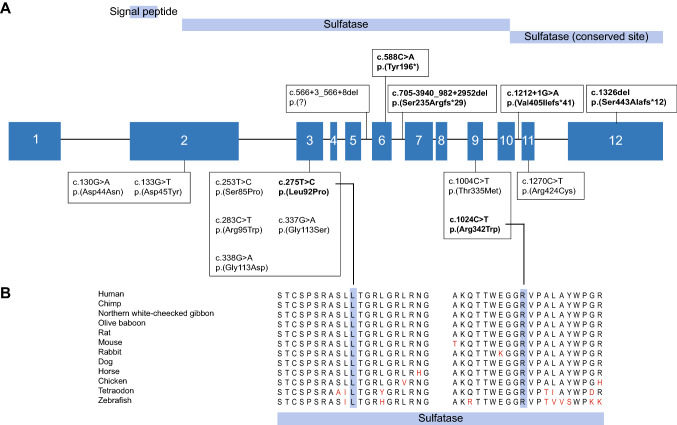
*ARSG* variants. **A** Schematic representation of the variability in location and type of novel and previously reported *ARSG* variants. The twelve *ARSG* exons are shown in dark blue squares. Protein domains are shown in light blue bars (Mistry et al. 2021). Variants that were identified in the present study are shown in bold. Truncating variants are presented above the exons, missense variants below. **B** Multiple sequence alignments of the sequence regions containing the two novel missense variants in 12 orthologs (Alamut Visual v.2.13). One-letter amino acid abbreviations are presented. Amino acids that do not correspond with the most conserved amino acid at their position are shown in red. The position of the variants are highlighted in blue. Both variants are located within the sulfatase protein domain

The genetic analyses of the three described cases revealed six heterozygous *ARSG* variants (Table 1, Fig. 2).

**Table 1:** Overview of *ARSG* variants of recruited subjects

Variant GRCh37 (hg19) NM_014960.5	Effect on RNA	Effect on protein NP_055775.2	GnomAD minor allele frequency (%)	Variant classification^a^	CADD_PHRED	Grantham score	Mutation taster	PhyloP	SIFT
Subject N									
Chr17:g.66391335G > A c.1212 + 1G > A	r.1212_1213ins[a;1212 + 2_1212 + 13]	p.(Val405Ilefs*41)	–	Pathogenic (PVS1-S, PS3-M, PM2, PM3, PP3, PP4)	34	N/a	N/a	6.163	N/a
Chr17:g.66339801T > C c.275T > C	r.(275u > c)	p.(Leu92Pro)	0.000398 / 0.008700 (Southern Europe)	Likely pathogenic (PS3-M, PM2, PM3, PP3, PP4)	27.5	98	Disease causing	6.704	Deleterious
Subject F									
Chr17:g.66416352del c.1326del	r.(1326del)	p.(Ser443Ala fs*12)	0.007781 / 0.025860 (Southern Europe)	Pathogenic (PVS1, PS3-M, PM2-Supp, PM3-Supp, PP4)	24.4	N/a	N/a	N/a	N/a
Chr17:g.66381246C > T c.1024C > T	r.1024c > u	p.(Arg342Trp)	0.000796 / 0.009930 (African/African American XX)	Likely pathogenic (PS3-M, PM2, PM3-Supp, PP3, PP4)	32	101	Disease causing	1.654	Deleterious
Subject D									
Chr17:g.66352829C > A c.588C > A	r.(588c > a)	p.(Tyr196*)	0.000396 / 0.002370 (North-western Europe)	Pathogenic (PVS1-S, PS3-M, PM2, PM3, PP4)	36	N/a	N/a	1.532	N/a
Chr17:g.66360749_66369617del c.705-3940_ 982 + 2952del	r.(705_982del)	p.(Ser235Arg fs*29)	-	Pathogenic (PVS1-S, PS3-M, PM2, PM3, PP4)	N/a	N/a	N/a	N/a	N/a

^a^Variant classification was performed using the American College of Medical Genetics and Genomics Standards and Guidelines (Abou Tayoun et al. 2018; Oza et al. 2018; Richards et al. 2015); CADD_PHRED, Combined Annotation Dependent Depletion, threshold value ≥ 15 (Kircher et al. 2014); ClinVar database (Landrum et al. 2020); GnomAD, Genome Aggregation Database, total population exome frequency/maximum exome frequency (corresponding population); Grantham score, threshold value ≥ 80 (Grantham 1974); MutationTaster (Schwarz et al. 2014); PhyloP, threshold value ≥ 2.7 (Pollard et al. 2010); SIFT, Sorting Intolerant From Tolerant (Ng and Henikoff 2001); N/a, not applicable; -, not present in database

In subject N, exome sequencing targeting gene panels for vision disorders and hearing impairment initially identified a heterozygous *SPP2* (OMIM 602637¸ isolated RP (Liu et al. 2015)) variant (NM_006944.3:c.439G > A NP_008875.1:p.(Glu147Lys)) and a possibly explanatory heterozygous *MCM2* (OMIM 116945, isolated SNHL OMIM 616968 (Gao et al. 2015)) variant (NM_004526.4:c.547C > T NP_004517.2:p.(Arg183Cys)). However, these variants were also identified in unaffected relatives (Supplemental Fig. 3). Re-analysis of the exome sequencing data with an updated gene panel for hearing impairment identified two variants in *ARSG*: NM_014960.5:c.1212 + 1G > A NP_055775.2:p.(?); NM_014960.5:c.275T > C NP_055775.2:p.(Leu92Pro). Segregation analysis showed that these variants were present in a compound heterozygous state (Supplemental Fig. 3) as there were no unaffected relatives with both *ARSG* variants. The c.275T > C p.(Leu92Pro) variant is a missense variant that was predicted to be pathogenic by all used *in-silico* prediction tools (Table 1). Skipping of exon 10 (out of frame) was predicted to be a consequence of the c.1212 + 1G > A p.(?) variant (SpliceAI: Donor splice site loss at −1nt (0.99/1) and acceptor splice site loss at −121nt (0.78/1)).

In subject F, a heterozygous variant (NM_206933.4:c.6524G > A NP_996816.3:p.(Arg2175His)) was identified in *USH2A* (OMIM 608400, USH and isolated RP OMIM 613809 (Rivolta et al. 2000)) and genome sequencing was performed to identify a second causal variant in this gene. However, no second *USH2A* variant could be identified and therefore variants in other USH-associated genes were addressed. This revealed two *ARSG* variants: NM_014960.5:c.1326del NP_055775.2:p.(Ser443Alafs*12); NM_014960.5:c.1024C > T NP_055775.2:p.(Arg342Trp). The c.1326del p.(Ser443Alafs*12) variant is a previously described pathogenic stop-gain variant (Peter et al. 2020). The c.1024C > T p.(Arg342Trp) variant is a missense variant that was predicted to be pathogenic by all used *in-silico* prediction tools (Table 1). In addition, this variant was predicted to result in skipping of exon 9 (out of frame) (SpliceAI: Donor splice site loss at + 67nt (0.15/1) and acceptor splice site loss at −41nt (0.25/1)). Segregation analysis could not be performed because relatives were not available.

Genome sequencing was initiated in subject D because no variants could be identified in genes associated with USH. Initially, four heterozygous variants in USH-associated genes were identified: a stop-gain variant in *ARSG* (NM_014960.5:c.588C > A NP_055775.2:p.(Tyr196*)), a non-canonical splice site variant in *ADGRV1* (OMIM 602,851, USH, NM_032119.4:c.5525-7C > T NP_115495.3:p.(?)), a missense variant in *MYO7A* (OMIM 276903, USH and isolated SNHL OMIM 601317 (Liu et al. 1997a, Liu et al. 1997b) and 600060 (Liu et al. 1997a, 1997b; Weil et al. 1997), NM_000260.4:c.905G > A NP_000251.3:p.(Arg302His)), and a deep-intronic variant in *PCDH15* (OMIM 605514, USH and isolated SNHL OMIM 609533 (Ahmed et al. 2003), NM_001142764.2:c.1098 + 2354G > A NP_001136236.1:p.(?)). The *in-silico* predictions for the missense variant in *MYO7A* were moderate (CADD_PHRED: 24.5 (threshold: 15), PhyloP: 2.5 (threshold: 2.7), Grantham: 29 (threshold: 80), MutationTaster: 'Benign' and SIFT: 'Tolerated') and the SpliceAI predictions for the *ADGRV1* and *PCDH15* variants were low (*ADGRV1*: acceptor splice site loss at + 11nt (0.01/1); *PCDH15:* acceptor splice site gain at + 124nt (0.24/1), acceptor splice site loss at −54nt (0.02/1), donor splice site gain at + 3nt (0.19/1)). Also, SpliceAI predicted no pseudo-exon inclusion for the *PCDH15* variant. Therefore, the *ARSG* variant was considered the strongest candidate in a gene associated with USH. Manual inspection of the sequencing reads (CRAM files) revealed indications for a deletion spanning exons 7 and 8 of *ARSG*. PCR and Sanger sequencing confirmed the deletion and breakpoints: NM_014960.5:c.705-3940_982 + 2952del NP_055775.2:p.(Ser235Argfs*29). PCR with a reverse primer designed to bind nucleotides deleted in the c.705-3940_982 + 2952del allele demonstrated that the c.588C > A p.(Tyr196*) variant and the deletion are in trans.

The identified *ARSG* variants were either not detected or detected at low frequencies (< 0.01%) in gnomAD (Table 1). The previously described variant (c.1326del p.(Ser443Alafs*12)) was classified as 'pathogenic' in the ClinVar database (Landrum et al. 2020) and according to the American College of Medical Genetics and Genomics (ACMG) Standards and Guidelines and its specifications for hearing loss (Table 1) (Abou Tayoun et al. 2018; Oza et al. 2018; Richards et al. 2015). Three novel variants were classified as 'pathogenic' and two as 'likely pathogenic' according to these guidelines. The identified missense variants affect amino acids within protein domains and that have been highly conserved throughout evolution (Fig. 2) (Mistry et al. 2021).

### Functional analyses of identified variants

**Fig. 3:**
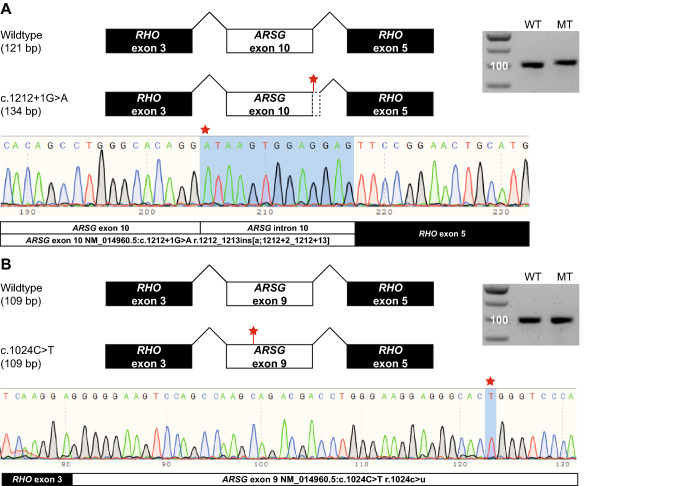
Results of minigene splice assays for *ARSG* variants c.1212 + 1G > A and c.1024C > T. In vitro splice assays were performed in HEK293T cells to validate the predicted splice defects. **A** A canonical splice site variant (c.1212 + 1G > A) was detected in *ARSG* (subject N) and was predicted by SpliceAI to cause skipping of exon 10 due to a donor splice site loss at −1nt (0.99/1) and acceptor splice site loss at −121nt (0.78/1). A minigene splice assay revealed the use of an alternative splice donor site at + 13nt in intron 10 which leads to an out of frame elongation of exon 10 (NM_014960.5:c.1212 + 1G > A r.1212_1213ins[a;1212 + 2_1212 + 13] NP_055775.2:p.(Val405Ilefs*41)). **B** A missense variant (c.1024C > T) was predicted by SpliceAI to cause skipping of exon 9 due to a donor splice site loss at + 67nt (0.15/1) and acceptor splice site loss at −41nt (0.25/1). A minigene splice assay did not confirm this and demonstrated no effect on splicing (NM_014960.5:c.1024C > T r.1024c > u NP_055775.2:p.(Arg342Trp)). *Bp* base pair; *WT* wildtype; *MT* mutant

**Fig. 4:**
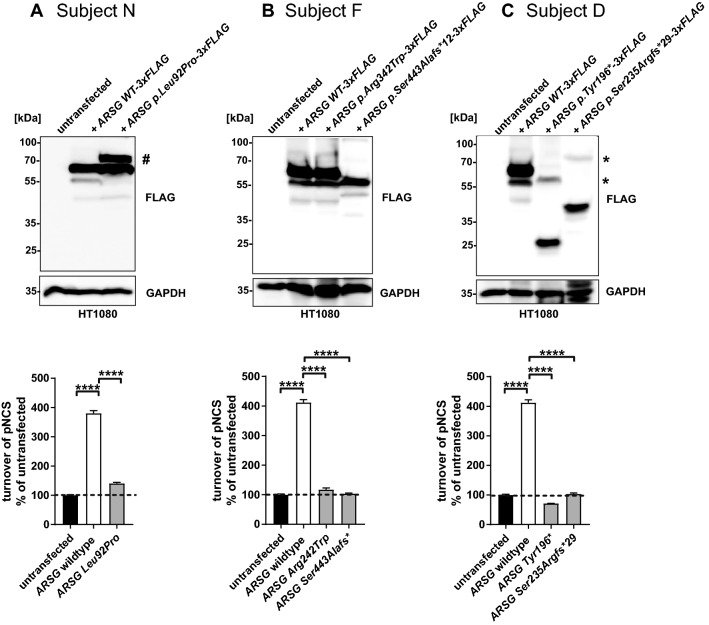
Functional analysis of *ARSG* variants identified in subjects N, F, and D. **A** Subject N: Immunoblot analysis of untransfected or stably transfected HT1080 cells with plasmids coding for 3xFLAG-tagged wildtype (WT) ARSG, along with the ARSG variant p.(Leu92Pro). An additional ARSG p.(Leu92Pro)-specific band is labeled with an #. Other bands in the figure are likely to be caused by limited proteolytic cleavage of ARSG. Total sulfatase activity of cell lysates from untransfected cells, or transfected cells expressing WT ARSG or ARSG with the pathogenic variant p.(Leu92Pro) against the artificial substrate P-nitrocatechol sulfate (pNCS). *N* = 3 replicates. Unpaired, two-tailed *t*-test, Mean ± SEM of the replicates. **B** Subject F: Immunoblot analysis of untransfected or stably transfected HT1080 cells with plasmids coding for 3xFLAG-tagged WT ARSG, along with the pathogenic variants p.(Arg342trp) and p.(Ser443Alafs*12). Other bands in the figure are likely to be caused by limited proteolytic cleavage of ARSG. Total sulfatase activity of cell lysates from untransfected cells, or from cells expressing WT ARSG or the pathogenic variants p.(Arg342Trp) and p.(Ser443Alafs*12) against the artificial substrate pNCS. *N* = 3 replicates. Unpaired, two-tailed *t*-test, Mean ± SEM of the replicates. **C** Subject D: Immunoblot analysis of untransfected or stably transfected HT1080 cells with plasmids coding for 3xFLAG-tagged WT ARSG, along with the pathogenic variants p.(Tyr196*) and p.(Ser235Argfs*29). High-molecular weight bands indicating possibly SDS-resistant dimers of the truncated proteins are labeled with an *. Other bands in the figure are likely to be caused by limited proteolytic cleavage of ARSG. Total sulfatase activity of cell lysates from untransfected cells, or cells expressing WT ARSG or the pathogenic variants p.(Tyr196*) and p.(Ser235Argfs*29) against the artificial substrate pNCS. *N* = 3 replicates. Unpaired, two-tailed *t*-test, Mean ± SEM of the replicates. Antibodies in all panels are against FLAG and GAPDH, used as a loading control. pNCS, P-nitrocatechol sulfate; SEM, standard error of the mean

The minigene splice assay for variant c.1212 + 1G > A (subject N), showed that the original splice donor site was lost, and that an alternative splice donor site at + 13nt in intron 10 was used. This resulted in an out of frame elongation of exon 10 and a premature stop codon (r.1212_1213ins[a;1212 + 2_1212 + 13] p.(Val405Ilefs*41) (Fig. 3). Stable expression in HT1080 fibrosarcoma cells of a construct bearing the second *ARSG* variant of subject N (c.275T > C p.(Leu92Pro)), followed by immunoblot analysis, revealed a modestly increased ARSG expression level compared to the WT construct (Fig. 4). An additional ARSG-specific band was detected for the p.(Leu92Pro) construct migrating at a higher apparent molecular weight. ARSG is extensively post-translationally modified by N-glycosylation and limited proteolysis (Kowalewski et al. 2014) and it could be speculated that the posttranslational modifications of this polypeptide differ, e.g., due retention in the endoplasmic reticulum. Notably, a minor band detected for the WT, most likely representing a proteolytically processed form migrating below the full-length protein, was nearly absent in the lane for the p.(Leu92Pro) construct. Sulfatase activity assays revealed a significant activity increase for the artificial substrate pNCS in cells stably expressing WT ARSG compared to untransfected cells. There was also a minimal increase in total sulfatase activity observed in cells expressing the p.(Leu92Pro) construct variant compared to untransfected cells, which, however, can likely be explained by the slightly higher expression (Fig. 4). This analysis indicates that the amino acid substitution presumably results in a nearly complete loss of enzyme activity.

Constructs encoding the truncated c.1326del p.(Ser443Alafs*12) ARSG (subject F) previously revealed similar ARSG expression levels as found in constructs encoding WT ARSG, but with a slightly lower molecular weight, in line with a truncated protein. Also, a significantly lower sulfatase activity was observed than for WT ARSG (Peter et al. 2020). These findings are confirmed in the present study in which we also found similar expression levels of the mutant construct as compared to WT ARSG with a slightly lower molecular weight, as well as significantly lower sulfatase activity in cells expressing the p.(Ser443Alafs*12) variant than in cells expressing WT ARSG (Fig. 4). Immunoblot analysis of ARSG with the amino acid substitution c.1024C > T p.(Arg342Trp) revealed that the protein is expressed at similar levels when compared to WT ARSG. Also, sulfatase activity showed no increase in the cells expressing the mutant ARSG compared to untransfected cells, indicating a complete loss of enzyme activity of the mutant ARSG. The minigene splice assay for this latter variant (c.1024C > T) revealed no effect on splicing (r.1024c > u p.(Arg342Trp)) (Fig. 3).

Finally, immunoblot analysis of the two truncated ARSG proteins c.588C > A p.(Tyr196*) and c.705-3940_982 + 2952del p.(Ser235Argfs*29) identified in subject D confirmed the truncated proteins to be of lower molecular weight than the WT ARSG, within their respective expected ranges (Fig. 4). Slightly lower signal intensities were observed for both mutant constructs compared to the WT construct, indicating lower expression levels of the mutant ARSG proteins. High-molecular weight bands were observed for both mutant constructs, indicating possibly SDS-resistant dimers of the truncated proteins. Determination of the sulfatase activity of cells expressing either mutant ARSG showed no increase of pNCS turnover compared to untransfected cells, indicating a complete loss of enzyme activity.

## Discussion

The present study confirms the recent introduction of USH type IV as being distinctive from the other three types of USH. We describe three subjects with an USH type IV phenotype caused by *ARSG* variants. This phenotype consists of late-onset RP and late-onset progressive SNHL. The retinal phenotype is characterized by a specific distribution of atrophy around the arcades, peripherally surrounded by intraretinal bone spicules best appreciated nasally and superior to the disc. There is progressive macular involvement eventually resulting in complete loss of the outer retina. There are no signs of vestibular dysfunction, although accurate testing to entirely exclude vestibular involvement could not be performed. In the three studied subjects, we identified six *ARSG* variants: one previously reported pathogenic single nucleotide deletion, three novel pathogenic variants, including the first reported structural *ARSG* variant, and two novel likely pathogenic missense variants (Table 1). Five identified variants were shown to cause loss of enzyme activity, in line with previous research (Khateb et al. 2018; Peter et al. 2020). For one variant, sulfatase was not tested, but this variant (c.1212 + 1G > A p.(Val405Ilefs*41)) is likely to result in loss of sulfatase activity since it leads to premature protein truncation. This is supported by the observation that the c.1212 + 1G > A p.(Val405Ilefs*41) variant occurs 38 amino acids N-terminal of the c.1326del p.(Ser443Alafs*12) variant, for which significantly reduced sulfatase activity was found in the current study (Fig. 4).

The lysosomal sulfatase ARSG is ubiquitously expressed in mice and has been detected the retina and the cochlea (Girotto et al. 2014; Kruszewski et al. 2016). An *A*rsg knockout (KO) mouse was shown to accumulate heparan sulfate in enlarged lysosomes and develop typical mucopolysaccharidosis-like features (Kowalewski et al. 2012). An early feature of the *Arsg* KO model is progressive photoreceptor degeneration in the retina and dysregulation of various lysosomal proteins, however, with no clear signs of lysosomal storage defects in retinal cells. Generally, these *Arsg* KO mice present with a milder phenotype than other mouse models of disorders resulting from impaired lysosomal degradation of heparan sulfate (Kruszewski et al. 2016). Immunohistochemistry of murine retina revealed ARSG expression in the retinal pigment epithelium (Kruszewski et al. 2016). A homozygous missense loss of function *ARSG* variant in a canine animal model resulted in sulfatase deficiency and neuronal ceroid lipofuscinosis, however, without affecting the retina (Abitbol et al. 2010). In mouse cochlea, ARSG was demonstrated to be located at the apical side of sensory hair cells in the organ of Corti (Girotto et al. 2014). In human, *ARSG* mRNA expression levels were the highest in the liver, kidney, pancreas and brain. Retina and cochlea were not included in these analyses (Ferrante et al. 2002; Frese et al. 2008). ARSG is ubiquitously expressed with the highest expression levels in the brain (Carithers et al. 2015; DeLuca et al. 2012; GTEx Consortium 2013, 2020; Melé et al. 2015). A major open question is how variants in *ARSG* cause USH type IV, and in particular how the impaired degradation of 3-*O*-modified heparan sulfate is causing cellular dysfunction and cell death despite no apparent lysosomal enlargement. Moreover, it is surprising that none of the patients reported so far show any signs of lysosomal storage defects in peripheral organs or central nervous system (CNS) involvement, as would be expected from the phenotype of the KO mice and ARSG-deficient dogs. Remarkably, a similar situation has been described for other genes causative for lysosomal storage diseases. While variants in genes associated with neuronal ceroid lipofuscinosis (*CLN3* (OMIM 607042), *CLN5* (OMIM 608102), and *MFSD8*/*CLN7* (OMIM611124)) typically lead to severe neuronal dysfunction and lysosomal storage in neurons and the peripheral tissues, some variants present with isolated retina disease (Bauwens et al. 2020; Khan et al. 2017; Ku et al. 2017; Magliyah et al. 2021). Similarly, variants in *HGSNAT* (OMIM 610453) are associated with mucopolysaccharidosis in the vast majority of patients, but isolated retina degeneration has also been described (Haer-Wigman et al. 2015). One could hypothesize that *ARSG* variants may also exhibit a broad spectrum of disease as well as isolated retina and cochlear defects. However, the variants we identified are all loss of function variants and lead to phenotypes of RP and SNHL, without neurological symptoms or signs of generalized clinical manifestations of lysosomal storage disease. Therefore, it is not expected that other variants would cause a more broad phenotype.

Comparing the twelve previously described cases supplemented with the presently identified subjects, we observed a fairly similar phenotype (Supplemental Table 3, 4) (Abad-Morales et al. 2020; Fowler et al. 2021; Igelman et al. 2021; Khateb et al. 2018; Peter et al. 2020). All individuals were diagnosed with RP with a midlife age of onset (35–60 years), which is later than generally seen in USH type I (first decade) (Tsilou et al. 2002; Van Camp and Smith 2020), type II (first or second decade) (Millan et al. 2011; Tsilou et al. 2002), or type III (variable but usually begins in the second decade) (Nisenbaum et al. 2021) (Fig. 1, Table 2). RP in the identified subjects is characterized by onset at the age of 40–60 years with unique but similar funduscopic changes. Funduscopic abnormalities were primarily seen around the arcades on initial examination. Atrophy was seen mainly around the arcades and extended into the mid-periphery with intraretinal bone-spicule hyperpigmentation adjacent to the atrophic border, most evident nasally and superior to the disc. The far periphery appeared normal. Fundus autofluorescence was consistent with the clinically visible changes and showed a typical pattern of hypoautofluorescence in the mid-periphery but with normal fundus autofluorescence in the far periphery. With age, the macula became more involved, resulting in complete loss of the outer retina in the fovea, as seen on OCT, leading to light perception vision. ERGs revealed lower scotopic than photopic responses, but eventually showed complete absence of both types of responses. All subjects identified in the present study and all but one previously described individuals suffered from progressive SNHL with a self-reported onset ranging from childhood to 50 years of age and an official diagnosis or the start of hearing aid use ranging from 18 to 67 years. Based on the PTA data, we calculated an age of onset of 17 years. Presumably, patients do not experience symptoms of their hearing loss until a more significant loss than the WHO cut-off value of 25 dB HL and thus have their hearing tested at a later age (Informal Working Group on Prevention of Deafness and Hearing Impairment Programme Planning & World Health Organization 1991). One individual reported no symptoms of SNHL. However, since no audiometric examination had been performed at his last visit at the age of 48, it could be that he was not yet aware of the possibly increased hearing thresholds or that this will manifest later in life (Igelman et al. 2021). The reported and calculated age of onset is obviously later than for USH types I and II (congenital), but also later than for type III with an age of onset of SNHL usually in the first decade of life (Fig. 1, Table 2) (Geng et al. 2017). Although, due to the limited age range of the subjects, we could only determine the age of onset of SNHL for USH type IV by interpolating the data. When comparing the USH IV ARTA with that of USH type Ib (Wagenaar et al. 1999), IIa (Hartel et al. 2016), and III (Plantinga et al. 2004) (Fig. 1), the difference in the severity of SNHL with USH Ib and the higher rate of progression compared to USH IIa are also notable.

**Table 2:** Suggested extension of clinical classification of Usher syndrome

	Hearing impairment	Visual impairment	Vestibular impairment
USH I	Congenital (Nisenbaum et al. 2021) Severe to profound (Nisenbaum et al. 2021) Stable	RP onset in first decade of life (Nisenbaum et al. 2021) Mean age of perceived night blindness: 10y (SD: 6.6) (Tsilou et al. 2002)	Severe (Nisenbaum et al. 2021)
USH II	Congenital (Nisenbaum et al. 2021) Moderate to severe (Nisenbaum et al. 2021) Variable progression (Stemerdink et al. 2021)	RP onset in second decade of life or later (Millan et al. 2011) Mean age of perceived night blindness: 15y (SD: 6.9) (Tsilou et al. 2002)	Uncertain (Stemerdink et al. 2021)
USH III	Variable age of onset, usually in first decade of life, range: childhood-35y (Geng et al. 2017) Progressive (Nisenbaum et al. 2021)	Variable RP onset, typically begin by second decade of life (Nisenbaum et al. 2021)	Variable (Nisenbaum et al. 2021)
USH IV	Range of self-reported age of onset: childhood-50y Range of official SNHL diagnosis or start of using hearing aids: 18–67y Moderate to severe	Range of RP onset: 30–60y	Uncertain (no patient-reported symptoms)

Classification of USH type I–III based on the original classification (Smith et al. 1994; Davenport and Omenn 1977), modified and expanded in response to recent findings. *y* years

It is noteworthy that two subjects (N and F) had a medical history of hypothyroidism, which was not reported in previous *ARSG* cases. Identification of larger numbers of USH type IV cases may answer the question on whether hypothyroidism is associated with *ARSG* defects.

Recent USH type IV reports expand the information on the variability of USH phenotypes. This aids in accurate patient counseling, a recurring challenge when patients are diagnosed with newly found or rarely described genotypes. In the literature, this considerable variability is evident in several reports of atypical USH phenotypes. First, some genes may be causative for both USH and isolated deafness or RP, for example *CDH23* (OMIM 605516, USH and isolated SNHL OMIM 601386 (Bork et al. 2001)). For that matter, there is no evidence of *ARSG* to be associated with non-syndromic RP or SNHL, neither in our in-house database (containing exome sequencing data of 2086 individuals analyzed for vision disorders and 2300 individuals analyzed for hearing impairment) nor in the literature to the best of our knowledge. Second, there are cases with variants in known USH-associated genes and phenotypes that differ from the corresponding type of USH, such as an USH II phenotype caused by *CDH23* variants or a phenotype with vestibular dysfunction caused by *USH2A* variants (Liu et al. 1999; Valero et al. 2019).

In conclusion, our observations confirm the newly described USH type IV caused by *ARSG* variants (Table 2). This type differs from the other USH types in the late onset of RP and SNHL. The retinal phenotype is characterized by a specific distribution of atrophy around the arcades, peripherally surrounded by intraretinal bone spicules best appreciated nasally and superior to the optic disc. There is progressive macular involvement, eventually resulting in complete loss of the central outer retina. Contrary to atypical USH, which we believe is an aberrant phenotype that results from variants in one of the previously identified USH genes, the *ARSG* phenotype is consistent and distinct from the original USH types I, II, and III. This study contributes to the determination of genotype–phenotype associations that are important not only for patient counseling, but also given the development of therapeutic strategies.

## Supplementary Information

Below is the link to the electronic supplementary material.

Supplementary file1 (DOCX 20 KB)

Supplementary file2 (DOCX 16 KB)

Supplementary file3 (DOCX 24 KB)

Supplementary file4 (DOCX 27 KB)

S Fig. 1 Retinal imaging of patient N. (**A**) Fundus photo (OD, at age 52 years) showing intraretinal bone-spicules predominantly located nasally and superior to the optic disc. (**B**) Fundus autofluorescence image (OD, at age 52 years) showing a ring of hyperautofluorescence in the posterior pole at the transition zone and confluent areas of hypoautofluorescence in the mid-periphery with preserved autofluorescence in the far periphery. (**C**) Optical coherence tomography (OD, at age 52 years) showing foveal sparing with only paracentral loss of the outer retina, corresponding to the relatively mild loss of visual acuity (OD 0.8 and OS 0.6 Snellen). (**D**) Goldmann perimetry (at age 49 years) showing an absolute ring scotoma with intact central vision and a relatively preserved peripheral field. OD, right eye; OS left eye. Supplementary file5 (EPS 8991 KB)

S Fig. 2 Available individual air conduction thresholds of included and previously described subjects. Subject F and N are reported in this study; subject LL64 (Peter 2020); subject MOL0120 III:2, MOL0737 II, and TB55 II (Khateb 2018). dB HL, decibel hearing level. Supplementary file6 (EPS 3432 KB)

S Fig. 3 Segregation analysis of subject N. The initially identified* SPP2* and* MCM2* variants were also found in unaffected relatives. The* ARSG* variants were shown to be present in a compound heterozygous state. *Subject II:1 was not tested. Supplementary file7 (EPS 770 KB)

## Data Availability

All data relevant to the study are included in the article or uploaded as supplementary information. The datasets generated and/or analyzed during the current study are available from the corresponding author upon reasonable request.

## Abbreviations

ARSG: Arylsulfatase G
bp: Base pair
dB HL: Decibel hearing level
ERG: Electroretinography
ER: Endoplasmic reticulum
HL: Hearing loss
KO: Knockout
OCT: Optical coherence tomography
pNCS: P-nitrocatechol sulfate
PTA: Pure-tone average
OD/OS: Right/left eye
RP: Retinitis pigmentosa
SNHL: Sensorineural hearing loss
USH: Usher syndrome
WT: Wild type
