# Supplementary material for: Usher syndrome type IV: clinically and molecularly confirmed by novel ARSG variants
Source: Hum Genet. 2022 Feb 28;141(11):1723–38. doi: 10.1007/s00439-022-02441-0 (PMC9556359; doi:10.1007/s00439-022-02441-0)
Supplement: Supplementary file 1 — Supplementary file1 (DOCX 20 KB) [file 439_2022_2441_MOESM1_ESM.docx]

**Supplemental table 1: Overview of previously reported *ARSG* variants associated with Usher syndrome.**

| **Report** | **Patient ID** | **Variant (allele 1;allele 2)** | **Reference sequence** |
| --- | --- | --- | --- |
| Khateb (Khateb 2018) | MOL0120 III:1, III:2; MOL0737 II:1, II:2; TB55 II:1 | c.133G>T p.(Asp45Tyr);c.133G>T p.(Asp45Tyr) | NM_014960.4 |
| Abad-Morales (Abad-Morales 2020) | N/a | c.130G>A p.(Asp44Asn);c.130G>A p.(Asp44Asn) | NM_014960.4 |
| Peter (Peter 2020) | LL64 | c.1326del p.(Ser443Alafs*12);c.1326del p.(Ser443Alafs*12) | NM_014960.4 |
|  | LL197 | c.253T>C p.(Ser85Pro);c.338G>A p.(Gly113Asp) | NM_014960.4 |
| Fowler (Fowler 2021) | N/a | c.1270C>T p.(Arg424Cys);c.1270C>T p.(Arg424Cys) | NR |
| Igelman (Igelman 2021) | ARSG-1 | c.283C>T p.(Arg95Trp);c.566+3_566+8del p.(?) | NR |
|  | ARSG-2 | c.1004C>T p.(Thr335Met);c.1326del p.(Ser443Alafs*12) | NR |
|  | ARSG-29692 | c.337G>A p.(Gly113Ser);c.337G>A p.(Gly113Ser) | NR |

N/a, not applicable; NR, not reported.
