# Supplementary material for: Usher syndrome type IV: clinically and molecularly confirmed by novel ARSG variants
Source: Hum Genet. 2022 Feb 28;141(11):1723–38. doi: 10.1007/s00439-022-02441-0 (PMC9556359; doi:10.1007/s00439-022-02441-0)
Supplement: Supplementary file 2 — Supplementary file2 (DOCX 16 KB) [file 439_2022_2441_MOESM2_ESM.docx]

**Supplemental table 2: Primer sequences used for functional analyses.**

| **Variant** | **Forward primer** | **Reverse primer** |
| --- | --- | --- |
| Immunoblot and sulfatase activity assay* | | |
| c.705-3940_982 +2952del | gtatgaattcatgggctggctttttctaaa (EcoRI sequence underlined) | gtatctcgagACTGGAACTCTGCCAGGCCAGTAAGCCAGTGCTGGGACCCGGTGCCCTCCTTCCCAGGTCGTCTGCTTGGCTGGACTTCCCCCTTGCACGCTGGATGAACT (XhoI sequence underlined) |
| c.588C>A | gtatgaattcatgggctggctttttctaaa (EcoRI sequence underlined) | gtatctcgagGTAACAGTCTCTTTGAAGGTT (XhoI sequence underlined) |
| c.1326del | gtatgaattcatgggctggctttttctaaag (EcoRI restriction site underlined) | gtatctcgagaaagctaatgctgcagctcaggccgcgcgcCCCATCACACGCCCTGGCTCC (XhoI restriction site underlined) |
| c.275T>C | gccggccggtgggcaaggaagcc | ggcttccttgcccaccggccggc |
| c.1024C>T | gtgctgggacccagtgccctccttc | gaaggagggcactgggtcccagcac |
| Minigene splice assay^#^ | | |
| c.1024C>T | GGGGACAAGTTTGTACAAAAAAGCAGGCTTCgtttccatcaagcatgacctc ( | GGGGACCACTTTGTACAAGAAAGCTGGGTGgtggacatctggactcctttc |
| c.1212+1G>A | GGGGACAAGTTTGTACAAAAAAGCAGGCTTCaacatcgcaagaccttgtctc | GGGGACCACTTTGTACAAGAAAGCTGGGTGgaactcggagctcaactcaac |

* Antisense sequence uppercase.

^#^ Gateway tail sequence in uppercase, *attB1* (forward primers) and *attB2* (reverse primers) site underlined, *ARSG* specific sequence lowercase.
