# Supplementary material for: Usher syndrome type IV: clinically and molecularly confirmed by novel ARSG variants
Source: Hum Genet. 2022 Feb 28;141(11):1723–38. doi: 10.1007/s00439-022-02441-0 (PMC9556359; doi:10.1007/s00439-022-02441-0)
Supplement: Supplementary file 3 — Supplementary file3 (DOCX 24 KB) [file 439_2022_2441_MOESM3_ESM.docx]

**Supplemental table 3: Overview of ophthalmic phenotype of (recruited) subjects.**

| **Patient ID** | **Sex**  (age at last exam) | **RP**  Age of onset | **Visual acuity** (age at exam) | **Visual fields** Age at and type of exam  Central scotoma; ring scotoma; peripheral visual field loss | **Retina** Macula; Mid-periphery; Periphery | **Autofluorescence (AF)** | | **Electroretinography (ERG)** | |
| --- | --- | --- | --- | --- | --- | --- | --- | --- | --- |
|  |  |  |  |  |  | HyperAF ring surrounding macular area | HypoAF at the level of the vascular arcades | Scotopic responses | Photopic responses |
| N | M (51y) | 46y | OD 20/25, OS 20/32 (49y) | 49y, GP  No; Yes; No | Paramacular atrophy; Pigmentation; Preserved | Yes | Yes | Moderate Decrease | Moderate decrease |
| F | F (72y) | 40y | OD HM, OS HM (71y) | 62y, GP  Yes; Yes; Yes | Atrophy; Pigmentation; Preserved | No | Yes | Severe decrease | Severe decrease |
| D | M (86y) | 51-60y | OD LP+, OS LP- (86y) | N/a  Self-reported peripheral visual field loss | Atrophy; Pigmentation; Preserved | No | Yes | N/a | N/a |
| MOL0120 III:1 (Khateb 2018) | M (70y) | 60y | OD LP+, OS LP+ (70y) | N/a | Para macular atrophy; Atrophy, dense pigmentation; Preserved | N/a | Yes | N/a | N/a |
| MOL0120 III:2 (Khateb 2018) | M (69y) | 55y | OD 20/100, OS HM (69y) | 65y, GP  No; Yes; No | Para macular atrophy; Atrophy, dense pigmentation; Preserved | Yes | Yes | Severe decrease | Moderate decrease |
| MOL0737 II:1 (Khateb 2018) | F (59y) | 45y | OD 20/100, OS 20/50 (59y) | 59y, GP  No; Yes; No | Preserved; Atrophy, cares pigmentation; Preserved | Yes | Yes | Moderate decrease | Mild decrease |
| MOL0737 II:2 (Khateb 2018) | F (55y) | 47y | OD 0.60, OS 20/25 (55y) | 55y, GP  No; Yes; No | Preserved; Atrophy, cares pigmentation; Preserved | Yes | Yes | Mild decrease | WNL |
| TB55 II:1 (Khateb 2018) | F (57y) | Late 40s | N/a | N/a | N/a | N/a | N/a | Severe decrease | Moderate decrease |
| Abad-Morales (Abad-Morales 2020) | F (40y) | 40y | OD 20/25, OS 20/25 (40y) | 40y, HFA  No; Yes; N/a | Preserved; Atrophy, cares pigmentation; Preserved | Yes | Yes | N/a | N/a |
| LL64 (Peter 2020) | F (72y) | 45y | OD 0.6, OS 20/40 (72y) | 69y, HFA  No; Yes; No | Preserved; Atrophy, cares pigmentation; Preserved | Yes | Yes | Severe decrease | Mild decrease |
| LL197 (Peter 2020) | F (59y) | 35y | OD HM, OS HM (59y) | 46y, GP  No; Yes; No | Para macular atrophy; Atrophy, scarce pigmentation; Preserved | Yes | Yes | Severe decrease | Mild decrease |
| Fowler (Fowler 2021) | M (60y) | 50y | OD 20/20, OS 20/25 (60y) | 60y, GP;  NR; Yes; No | Perimacular atrophy; Pigmentation; Preserved | Yes | Yes | N/a | N/a |
| ARSG-1 (Igelman 2021) | M (48y) | Early 30s | OD 20/20, OS 20/25 (40y) | N/a | No findings; N/a; N/a | N/a | N/a | N/a | N/a |
| ARSG-2 (Igelman 2021) | F (65y) | 65y | OD 20/30, OS 20/25 (65y) | N/a | Foveal sparing atrophy; N/a; N/a | N/a | N/a | Moderate decrease | Moderate decrease |
| ARSG-29692 (Igelman 2021) | F (69y) | 40y | OD 20/1000, OS 20/800 (69y) | N/a | Atrophy; N/a; N/a | N/a | N/a | N/a | N/a |

GP, Goldmann perimetry; HFA, Humphrey field analyzer; HM, hand motion; LP, light perception; N/a, not applicable; NR, not reported; OD; right eye; OS; left eye; y, years.
