# Supplementary material for: Usher syndrome type IV: clinically and molecularly confirmed by novel ARSG variants
Source: Hum Genet. 2022 Feb 28;141(11):1723–38. doi: 10.1007/s00439-022-02441-0 (PMC9556359; doi:10.1007/s00439-022-02441-0)
Supplement: Supplementary file 4 — Supplementary file4 (DOCX 27 KB) [file 439_2022_2441_MOESM4_ESM.docx]

**Supplemental table 4: Overview of audiovestibular phenotype of (recruited) subjects.**

| **Patient ID** | **Sex**  (age at last exam) | **SNHL**  Age of onset | **Audiogram**  Age; configuration | **PTA_0.5-4 kHz_**  R / L | **SRT**  R / L | **Vestibular dysfunction** |
| --- | --- | --- | --- | --- | --- | --- |
| N | M (51y) | 42y^b^ | 46y; 49y; Descending | 46 / 53 dB HL; 51 / 55 dB HL (bone conduction) | N/a | No |
| F | F (72y) | 20y^a^ | 60y 72y; Descending | 72.5 / 72.5 dB HL; 90 / 85 dB HL | N/a | No |
| D | M (86y) | 31-40y^a^ | N/a | | | No |
| MOL0120 III:1 (Khateb 2018) | M (69y) | 67y^b^ | N/a; Descending | | 65 / 60 dB HL | No |
| MOL0120 III:2 (Khateb 2018) | M (70y) | 45-48y^a^ | 57y; Descending | 61.5 / 62.5 dB HL | 50 / 50 dB HL | No |
| MOL0737 II:1 (Khateb 2018) | F (59y) | 48y^b^ | 58y; Flat | 67.5 / 69 dB HL | 60 / 60 dB HL | No |
| MOL0737 II:2 (Khateb 2018) | F (55y) | 35y^b^ | 55y; Flat | 72.5 / 75 dB HL | 65 / 65 dB HL | No |
| TB55 II:1 (Khateb 2018) | F (57y) | Childhood^a^, 18y^b^ | 56y; Flat | 90 / 75 dB HL | 70 / 80 dB HL | No |
| Abad-Morales (Abad-Morales 2020) | F (40y) | Infancy^a^, 40-44y^b^ | N/a; Descending | | 50 / 40 dB HL | No |
| LL64 (Peter 2020) | F (72y) | 40y^a^, 67y^b^ | 67y; Descending | 74 / 69 dB HL |  | No |
| LL197 (Peter 2020) | F (59y) | 50y^a^ | N/a | | | No |
| Fowler (Fowler 2021) | M (60y) | 40y^a^ | N/a | | | No |
| ARSG-1 (Igelman 2021) | M (48y) | No | N/a | | | No |
| ARSG-2 (Igelman 2021) | F (65y) | 50y^a^ (unclear progression) | N/a | | | No |
| ARSG-29692 (Igelman 2021) | F (69y) | 50y^a^ (stable) | N/a | | | Yes |

SNHL refers to progressive, moderate to severe SNHL unless otherwise stated. PTAs are air conduction PTAs unless otherwise stated. ^a^Self-reported start of symptoms. ^b^Age of official SNHL diagnosis or start of using hearing aids. dB HL, decibel hearing level; L, left; N/a, not available; PTA, pure tone average; R, right; SNHL, sensorineural hearing loss; SRT, speech recognition threshold; y, years.
